# Case of Strangulated Hernia, with Adhesions to the Sac

**Published:** 1834-07-01

**Authors:** John Valentine


### *Case of Strangulated Hernia, with Adhesions to the Sac.*
By JOHN VALENTINE, M.R.C.S.

ON Monday, April 20th, 1829, I was called to Jonas Pyke, ætat. seventy-two, residing at West Charlton, three miles distant from this town. On examination, I found he had a scrotal hernia, with which he had been afflicted the last forty years. It appeared to be formed of omentum and intestine, and had been partly reducible at times, but now was altogether strangulated, attended with constipation, tenderness of the abdomen, &c.; which symptoms had existed ever since the preceding Saturday. I immediately attempted to reduce it by the taxis, but failed. His pulse being full and hard, I was induced to bleed him *ad syncopen*, in which state I also attempted the taxis, but again failed. Under these circumstances, and considering the length of time the strangulation had existed, I proposed the operation, which being assented to, I performed it in about half an hour after.

Having opened the sac, I found a considerable quantity of omentum and mesentery completely agglutinated, and adhering in every direction to it. I then made a small incision at the bottom of the mass, introduced a director, and laid it open up to the ring, which exposed a knuckle of intestine, (the ileum,) of a dark brown colour, which I endeavoured to replace, but could not effect it. I then, by means of a common probe-pointed bistoury, with a gently conducted sawing motion, divided the stricture at the external ring; after which the intestine was easily reduced. Reflecting on the advanced age of the patient, and the circumstance of the hernia not being ever wholly reducible, (at least for many years,) I brought the edges of the incision together, without cutting off the omentum, or returning it into the abdomen; a practice occasionally adopted by some eminent surgeons. The poor old man bore the operation well, and expressed himself relieved by it; he was placed in bed, and small doses of castor-oil were given him at intervals.

I visited him the next day, and was informed that he had had two motions, but still complained of tenderness of the abdomen; his pulse was strong, and rather full. I took away a pint of blood, and directed ten leeches to be applied on the right side, just above the incision; which measures had the best effect, and he had not an unpleasant symptom after. The wound healed in a very short time; he perfectly recovered, and is now living.

Mr. Brodie, in one of his clinical lectures, sanctions the above practice. He is reported to have said, that excision of the omentum ought not to be performed in all cases; and that, in cases where there is not a very large quantity of omentum in the hernia, but where it is extensively adherent to the surface of the sac, it is safer to leave it where it is found, than to cut off a portion of it, or to dissect through the adhesions. The success of this practice, in two cases which he relates, is certainly in its favour. The patients are left as well off as they were before the strangulation took place, and probably better; inasmuch as it is most likely that the omentum, after the operation, must have contracted adhesions to the hernial sac, making them less liable to a descent of the intestine.

The sentiments of the late Mr. Hey coincide with those of Mr. Brodie. In his valuable work on Surgery are the following remarks on the subject in question.

"When the portion of omentum which is prolapsed is in a sound state, of little bulk, and strongly adherent to the hernial sac; and when, from inquiries made of the patient, we learn that this small part has been prolapsed for many years, without disturbing the functions of the abdominal viscera, we may fairly conclude that we shall not injure those functions by leaving such a portion in its prolapsed state. In such a case I have suffered the omentum to remain, and have found no difficulty in healing the wound, nor any injury afterwards from the application of a well-adapted truss. In one patient I left a portion, which I judged to be about two ounces avoirdupois in weight, which was the largest portion that I have suffered to remain. The wound was healed at the expiration of six weeks after the operation." (*Practical Observ. in Surgery*, p. 171.)

*Somerton, Somersetshire; June* 10, 1834.

